# Identification of Novel Genomic Regions for Biofortification Traits Using an SNP Marker-Enriched Linkage Map in Wheat (*Triticum aestivum* L.)

**DOI:** 10.3389/fnut.2021.669444

**Published:** 2021-06-15

**Authors:** Gopalareddy Krishnappa, Nagenahalli Dharmegowda Rathan, Deepmala Sehgal, Arvind Kumar Ahlawat, Santosh Kumar Singh, Sumit Kumar Singh, Ram Bihari Shukla, Jai Prakash Jaiswal, Ishwar Singh Solanki, Gyanendra Pratap Singh, Anju Mahendru Singh

**Affiliations:** ^1^Division of Genetics, Indian Council of Agricultural Research-Indian Agricultural Research Institute, New Delhi, India; ^2^Division of Crop Improvement, Indian Council of Agricultural Research-Indian Institute of Wheat and Barley Research, Karnal, India; ^3^International Maize and Wheat Improvement Center, Texcoco, Mexico; ^4^Department of Genetics and Plant Breeding, Govind Ballabh Pant University of Agriculture and Technology, Pantnagar, India; ^5^Indian Council of Agricultural Research-Indian Agricultural Research Institute, Regional Station, Samastipur, India

**Keywords:** biofortification, QTLs, malnutrition, SNPs, SSRs, mapping

## Abstract

Micronutrient and protein malnutrition is recognized among the major global health issues. Genetic biofortification is a cost-effective and sustainable strategy to tackle malnutrition. Genomic regions governing grain iron concentration (GFeC), grain zinc concentration (GZnC), grain protein content (GPC), and thousand kernel weight (TKW) were investigated in a set of 163 recombinant inbred lines (RILs) derived from a cross between cultivated wheat variety WH542 and a synthetic derivative (*Triticum dicoccon* PI94624/*Aegilops tauschii* [409]//BCN). The RIL population was genotyped using 100 simple-sequence repeat (SSR) and 736 single nucleotide polymorphism (SNP) markers and phenotyped in six environments. The constructed genetic map had a total genetic length of 7,057 cM. A total of 21 novel quantitative trait loci (QTL) were identified in 13 chromosomes representing all three genomes of wheat. The trait-wise highest number of QTL was identified for GPC (10 QTL), followed by GZnC (six QTL), GFeC (three QTL), and TKW (two QTL). Four novel stable QTL (*QGFe.iari-7D.1, QGFe.iari-7D.2, QGPC.iari-7D.2*, and *QTkw.iari-7D*) were identified in two or more environments. Two novel pleiotropic genomic regions falling between *Xgwm350–AX-94958668* and *Xwmc550–Xgwm350* in chromosome 7D harboring co-localized QTL governing two or more traits were also identified. The identified novel QTL, particularly stable and co-localized QTL, will be validated to estimate their effects on different genetic backgrounds for subsequent use in marker-assisted selection (MAS). Best QTL combinations were identified by the estimation of additive effects of the stable QTL for GFeC, GZnC, and GPC. A total of 11 RILs (eight for GZnC and three for GPC) having favorable QTL combinations identified in this study can be used as potential donors to develop bread wheat varieties with enhanced micronutrients and protein.

## Introduction

Wheat (*Triticum* spp.) is a major staple cereal crop contributing about 20% calories to the diet and at least 30% of Fe and Zn intake worldwide. Even though it has the highest levels of micronutrients among the three major cereals viz., wheat, rice, and maize, most wheat-based diets fail to deliver the required quantity of essential nutrients, such as iron and zinc. Malnutrition due to insufficient intake of micronutrients, such as iron and zinc, has been recognized as one of the major global health issues affecting nearly three billion people across the globe. The intensity of the risk is high in nations dominated by cereal-based diets (1). Around 25% of the global population is affected by anemia because of Fe deficiency (2), and the leading risk groups for this global public health concern are children 0–5 years of age, and pregnant and lactating women. Anemic complex due to severe iron deficiency leads to several life-threatening diseases, namely, chronic kidney and heart failure, and inflammatory bowel disease (3).

Zinc is an essential element for a wide range of biochemical and immunological functions, and acute zinc deficiency leads to major health difficulties, such as altered growth and development, immunity, pregnancy, and neurobehavioral adversities (4). Estimates indicate that around 17% of the global population suffers from zinc deficiency-related diseases (5). Grain protein quantity and quality determine both the nutritional and end-product quality of wheat. Lack of secondary immunity due to protein energy malnutrition (PEM) is one of the common causes of several infections in humans. Acute PEM in children is clinically defined as marasmus (chronic wasting) or kwashiorkor (edema and anemia) (6). Chronic PEM in children results in impaired cognitive development (7). Micronutrient malnutrition and PEM are leading risk factors for health loss in developing countries, with pregnant women and young children forming the most vulnerable groups (8).

Micronutrient and protein malnutrition can be overcome by consuming nutrient-rich diverse diet and/or by supplementation and fortification. However, the majority of populations in which the malnutrition problem is alarming may not be able to afford either of the two options, particularly the remote rural poor (9). Moreover, these interventions are not sustainable. Enhancing the nutritive levels of crop plants by conventional and molecular breeding approaches, termed as “biofortification,” has been recognized as a cost-effective and sustainable approach to reduce global protein and micronutrient malnutrition. Currently, the development of biofortified crop varieties in many countries has gained momentum, particularly after reaching self-sufficiency in food grains.

Grain mineral density depends on a plethora of physiological and biochemical processes, such as mineral absorption, translocation, redistribution, and remobilization to the sink, which makes micronutrient accumulation in grain a complex trait (10). Therefore, breeding programs need to be re-oriented to broaden the genetic base using wild relatives and landraces, and dissecting the genetic basis of these nutritional quality traits (11). Landraces are one of the most important sources of wheat biofortification with high levels of micronutrients (12). Conventional breeding approaches have been successfully used to incorporate higher grain zinc content into elite breeding materials by crossing high-yielding elite wheat lines with *A tauschii*-based synthetic hexaploid wheats or *Triticum spelta* accessions (13). Substitution lines of the 6B chromosome obtained from *Triticum dicoccoides* are one of the most common genetic resources to improve zinc concentration in wheat (14). The *Gpc-B1* locus mapped on the short arm of the 6B chromosome, derived from *T dicoccoides*, has a pleiotropic effect on zinc and iron in addition to grain protein (15). An NAC transcription factor (NAM-B1) encoded by *Gpc-B1* is responsible for the increase in zinc as well as iron levels, possibly by stimulating leaf senescence, and thus remobilization of zinc and iron from flag leaves into seeds (16). Synthetic wheat derived from *Ae. tauschii* contains higher grain zinc and can act as a valuable genetic resource to increase the grain zinc levels of cultivated wheat (17).

Genetic dissection of complex nutritional traits is important for their improvement through marker-assisted selection (MAS). Identification of tightly linked molecular markers to the genomic regions governing the traits would help in the improvement of otherwise difficult to breed complex traits like protein and micronutrients. Reports have indicated significant effects of the environment and genotype-by-environment interaction (GEI) in the expression of PC and TKW (18–23), iron, and zinc (19, 24, 25). Molecular mapping of polygenic traits by identifying quantitative trait loci (QTL) harboring genes for protein, micronutrient, and TKW would allow plant breeders to more efficiently develop biofortified cultivars.

QTL have been identified for grain iron (19, 26–38), grain zinc (13, 19, 27–33, 35–41), grain protein content (19, 27, 31, 35, 36, 42–50), and thousand kernel weight (19, 27, 29, 45, 51–54). However, most investigations on mapping nutritional quality have exploited low-density maps, which have resulted in large interval QTL that have rarely been exploited in breeding.

Previous mapping of the same RIL population was carried out with 136 polymorphic SSR markers, which led to the identification of 16 QTL for four traits (55). The linkage map was coarse because of low marker frequency per chromosome ranging from 6 (1A and 2A) to 11 markers per chromosome (7B). Also, no QTL were mapped on the D genome because of low marker coverage. In this study, a 35K SNP chip was used for genotyping the RIL population, and a combined dataset of SSR and SNP markers was used to identify QTL for nutritional traits.

## Materials and Methods

### Plant Material

A set of 286 RILs from a cross between Indian bread wheat variety WH 542 and a synthetic derivative (*T. dicoccon* PI94624/*Ae. tauschii* [409]//BCN) received from CIMMYT (International Maize and Wheat Improvement Center), Mexico, was used in the earlier mapping study with SSR markers (55). A subset of 163 randomly selected RILs from this population was used for this investigation.

### Field Trials and Phenotyping

The details of field experimentation, sample collection, and phenotyping have been described in detail in the earlier study (55). The phenotypic data for GFeC, GZnC, GPC, and TKW recorded for the earlier study were converted into the best linear unbiased predictors (BLUPs) and used in this study. Phenotypic correlations among traits, heritability, and ANOVA were conducted using the MetaRv6.0 (Multi Environment Trial Analysis with R) software. BLUPs of each RIL obtained for an individual year and combined across years were used further in QTL analysis. Phenotypic data of all the six environments are presented as Supplementary Table 1.

### Genotyping

RILs and parental genomic DNA were extracted from the leaves of 21-day-old seedlings by following the CTAB method of Murray and Thompson (56).

#### Genotyping With Single Nucleotide Polymorphism Markers

The 163 RILs and parental lines were genotyped using Axiom Wheat Breeder's Genotyping Array (Affymetrix, Santa Clara, CA, United States) with 35,143 SNPs (https://www.cerealsdb.uk.net).

#### Genotyping With Simple-Sequence Repeat Markers

A total of 714 SSR markers (57, 58) were used for the parental polymorphism survey. These selected 714 SSRs cover all the chromosome arms of the bread wheat genome. Polymorphic markers and genotypic data are presented as Supplementary Table 2.

### Linkage Analysis and Quantitative Trait Locus Mapping

Monomorphic markers between the two parents and markers with more than 30% missing data and minor allele frequency ≤ 5 and ≥95% were eliminated. Furthermore, markers that showed significant segregation distortion (*p* < 0.0001) from the expected 1:1 ratio and redundant markers were discarded using bin function in QTL ICIM Mapping v4.2. Finally, a high-quality filtered set of 836 informative markers (736 SNPs + 100 SSRs) was utilized for the QTL analysis.

Both linkage and QTL analysis were conducted with the IciMapping v4.2 software (http://www.isbreeding.net). The chromosome location of SNP inferred by BLAST of the sequences and previously mapped SSR markers (55) was used as the anchoring information. A LOD threshold of 3 was specified for grouping the markers. After all the markers were correctly grouped, they were ordered using the k-Optimality algorithm. Then, Rippling was done to fine-tune the ordered chromosomes in the linkage groups using a 5 cM window size. ICIM-ADD method was employed, which conducts inclusive composite interval mapping for identifying QTL. Missing phenotypic data were considered as deletion during QTL mapping and a relaxed threshold LOD score of 2.5 was specified for declaring significant QTL.

### *In silico* Analysis of Quantitative Trait Loci

An *in silico* search of candidate genes was performed in the Ensemble Plants database (http://plants.ensembl.org/index.html) of the bread wheat genome with the Basic Local Alignment Search Tool (BLAST) using default parameters. The sequences of the markers present within the peak of the QTL and the flanking markers were used to conduct the search.

## Results

### Variability, Heritability, and Trait Correlations

**Figure 1:**
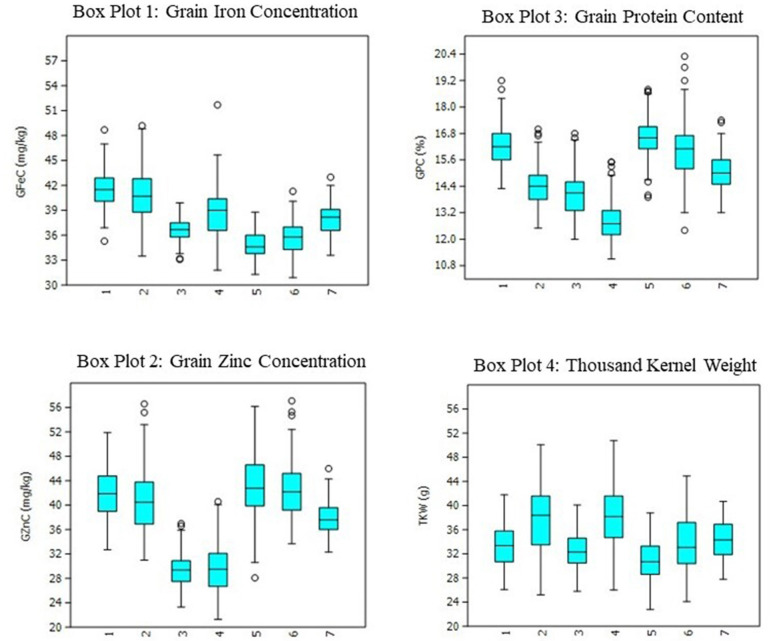
Boxplots for grain iron, zinc, protein, and thousand kernel weight in RIL population grown across three locations for 2 years. 1: ICAR-IARI_Y1; 2: ICAR-IARI_Y2; 3: GBPUA&T_Y1; 4: GBPUA&T_Y2: 5: Pusa Bihar_Y1; 6: Pusa Bihar_Y2; 7: Pooled mean.

**Figure 2:**
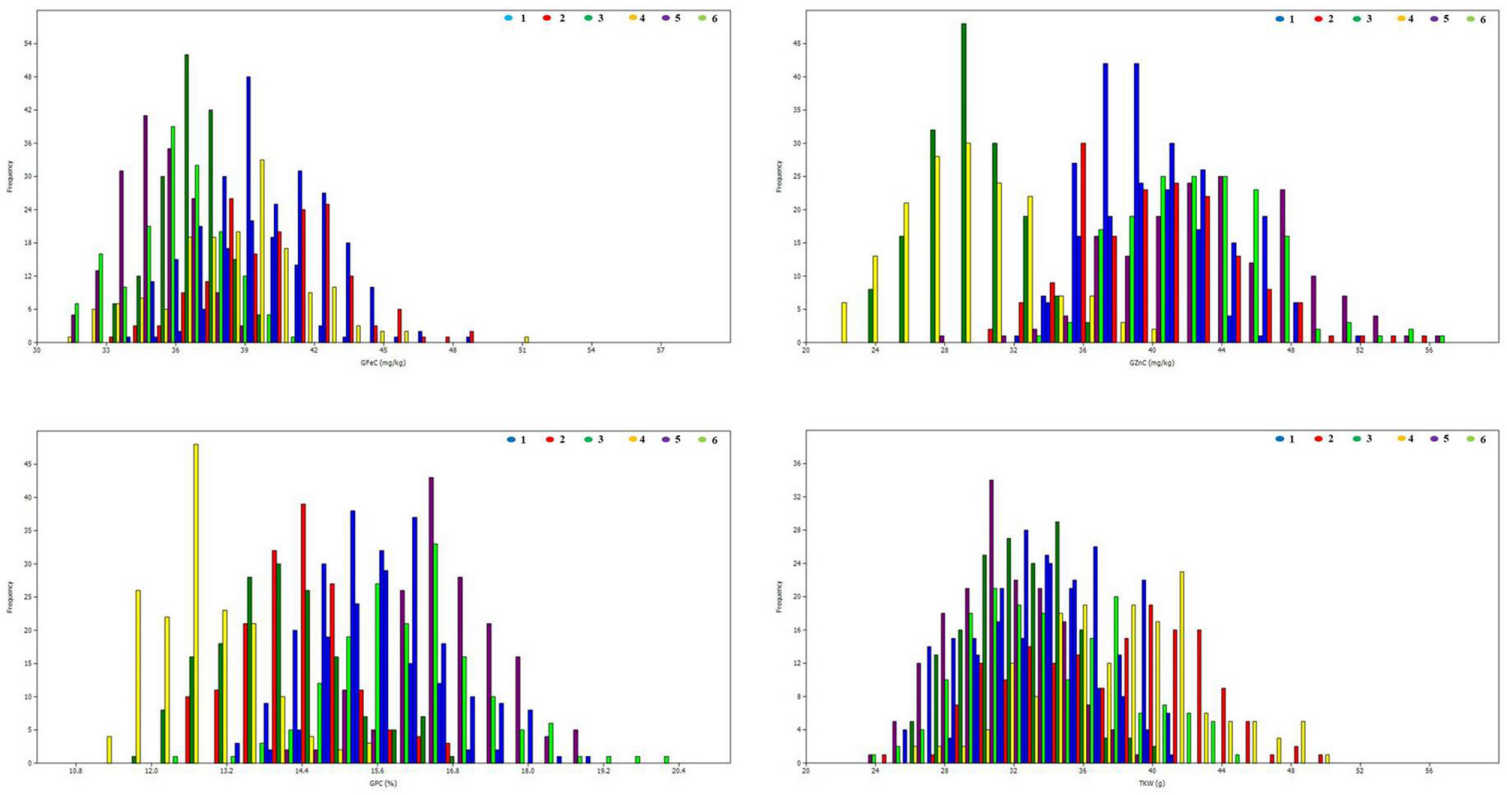
Frequency distributions for grain iron, zinc, protein, and thousand kernel weight in RIL population grown across three locations for 2 years. 1: ICAR-IARI_2012–13; 2: ICAR-IARI_2013–14; 3: GBPUA&T_2012–13; 4: GBPUA&T_2013–14: 5: Pusa Bihar_2012–13; 6: Pusa Bihar_2013–14.

The heritability and variance components of GFeC, GZnC, GPC, and TKW in a RIL population are presented in Table 1. Parents were contrasting for all the studied traits and P2 was superior over P1 with 43, 31, 26, and 23%, respectively, for GFeC, GZnC, GPC, and TKW. Environment-wise heritability ranged from 0.54 (GFeC at GBPUA&T_Y1) to 0.96 (TKW at ICAR-IARI_Y2 and GBPUA&T_Y2) across the traits. The lowest pooled heritability was observed for GZnC (0.77), whereas, highest pooled heritability was recorded for TKW (0.91). Trait heritability corroborates the variance components; GZnC (6.87%) and TKW (4.57%) recorded the highest and lowest CV, respectively. The genotypic variance was highly significant for all the studied traits across the environments. The environment-wise pooled mean is also represented graphically in Figure 1. All the studied traits exhibited a near-normal distribution (Figure 2). Genetic correlation coefficients among GFeC, GZnC, GPC, and TKW are presented in Table 2. All the associations among the studied traits are positive and significant, except, between TKW and GPC in the Pusa Bihar_Y1 (r_g_ = −0.03) and Pusa Bihar_Y2 environments (0.1) (Table 2).

**Table 1:** Heritability and variance components of grain iron, zinc, protein, and thousand kernel weight in RIL population grown across three locations for 2 years.

**Trait**	**Environment**	**Parental mean**		**RIL population h**^****2****^ **(bs) and variance**			
		**WH542 (P1)**	**Synthetic derivative (P2)**	**h^**2**^ (bs)**	**Genotype Variance**	**LSD**	**CV%**
Grain iron (ppm)	ICAR-IARI_Y1	33.8	49.6	0.76	6.44^***^	3.49	4.90
	ICAR-IARI_Y2	33.8	45.0	0.78	10.30^***^	4.20	5.90
	GBPUA&T_Y1	30.3	45.8	0.54	3.46^***^	3.54	6.67
	GBPUA&T_Y2	32.1	42.0	0.82	11.98^***^	4.10	5.92
	Pusa Bihar_Y1	30.0	45.2	0.66	3.76^***^	3.16	5.66
	Pusa Bihar_Y2	30.0	44.7	0.72	5.93^***^	3.62	6.08
	Pooled mean	31.7	45.4	0.81	3.93^***^	2.41	5.86
Grain zinc (ppm)	ICAR-IARI_Y1	37.7	48.8	0.81	17.98^***^	5.10	6.84
	ICAR-IARI_Y2	37.2	52.3	0.87	23.91^***^	4.85	6.44
	GBPUA&T_Y1	27.7	39.3	0.73	9.81^***^	4.51	9.05
	GBPUA&T_Y2	26.9	38.5	0.86	17.75^***^	4.42	8.19
	Pusa Bihar_Y1	39.8	45.9	0.90	26.57^***^	4.55	5.65
	Pusa Bihar_Y2	41.1	51.0	0.86	20.56^***^	4.69	6.05
	Pooled mean	35.1	46.0	0.77	8.28^***^	3.81	6.87
Grain protein	ICAR-IARI_Y1	14.6	18.6	0.78	1.21^***^	1.43	5.04
content (%)	ICAR-IARI_Y2	12.4	18.5	0.65	1.17^***^	1.78	7.74
	GBPUA&T_Y1	12.8	16.3	0.78	1.40^***^	1.54	6.24
	GBPUA&T_Y2	11.3	14.6	0.67	1.11^***^	1.69	8.16
	Pusa Bihar_Y1	15.9	19.1	0.78	1.08^***^	1.35	4.64
	Pusa Bihar_Y2	15.7	17.8	0.78	1.88^***^	1.80	6.45
	Pooled mean	13.8	17.5	0.84	0.81^***^	1.01	6.34
Thousand kernel	ICAR-IARI_Y1	28.1	35.0	0.92	12.93^***^	2.83	4.49
weight (gm)	ICAR-IARI_Y2	29.1	36.9	0.96	27.32^***^	3.00	4.10
	GBPUA&T_Y1	29.6	35.1	0.91	9.96^***^	2.66	4.36
	GBPUA&T_Y2	31.2	33.8	0.96	25.01^***^	2.94	3.99
	Pusa Bihar_Y1	26.8	32.7	0.89	10.57^***^	2.94	5.09
	Pusa Bihar_Y2	25.1	35.9	0.93	21.35^***^	3.49	5.44
	Pooled mean	28.3	34.9	0.91	11.99^***^	2.91	4.57

*** *Significant at p < 0.001; Y1: 2012–13; Y2: 2013–14; P1: WH542; P2: synthetic derivative; h^2^ (bs), heritability (broad sense); LSD, least significant difference; CV, coefficient of variation*.

**Table 2:** Genetic correlation coefficients among grain iron, zinc, protein, and thousand kernel weight in RIL population grown across three locations for 2 years.

	**Traits**	**GFeC**	**GZnC**	**GPC**
ICAR-IARI_Y1	GZnC	0.65^***^		
	GPC	0.68^***^	0.61^***^	
	TKW	0.55^***^	0.43^***^	0.24^**^
ICAR-IARI_Y2	GZnC	0.64^***^		
	GPC	0.76^***^	0.56^***^	
	TKW	0.58^***^	0.36^***^	0.46^***^
GBPUAT_Y1	GZnC	0.73^***^		
	GPC	0.54^***^	0.34^***^	
	TKW	0.62^***^	0.52^***^	0.21^**^
GBPUAT_Y2	GZnC	0.45^***^		
	GPC	0.27^***^	0.10	
	TKW	0.50^***^	0.37^***^	0.27^***^
Pusa Bihar_Y1	GZnC	0.48^***^		
	GPC	0.53^***^	0.37^***^	
	TKW	0.50^***^	0.21^**^	−0.03
Pusa Bihar_Y2	GZnC	0.38^***^		
	GPC	0.55^***^	0.32^***^	
	TKW	0.51^***^	0.34^***^	0.10
Pooled mean	GZnC	0.97^***^		
	GPC	0.95^***^	0.57^***^	
	TKW	0.81^***^	0.94^***^	0.52^***^

** *Significant at p < 0.01;*

*** *significant at p < 0.001; Y1: 2012–13; Y2: 2013–14; GZnC, grain zinc concentration; GFeC, grain iron concentration; GPC, grain protein content; TKW, thousand kernel weight*.

### Quantitative Trait Locus Mapping

The total genetic length of the linkage map was 7,057 cM, and it contained 736 SNPs and 100 SSRs. The chromosome and genome-wise distribution of markers is presented in Table 3. The B genome had the highest number of mapped markers (361) followed by the A (265) and D genomes (210). Chromosome-wise distribution of the markers ranged between 17 (6A chromosome) to 81 (7A chromosome).

**Table 3:** Number of markers grouped by each wheat chromosome and genome in the RIL mapping population.

**Chromosome**	***Triticum aestivum*** **genome**		
	**A**	**B**	**D**
Chromosome 1	48	26	31
Chromosome 2	20	63	22
Chromosome 3	21	64	25
Chromosome 4	54	50	33
Chromosome 5	24	61	50
Chromosome 6	17	34	27
Chromosome 7	81	63	22
Total	265	361	210

**Figure 3:**
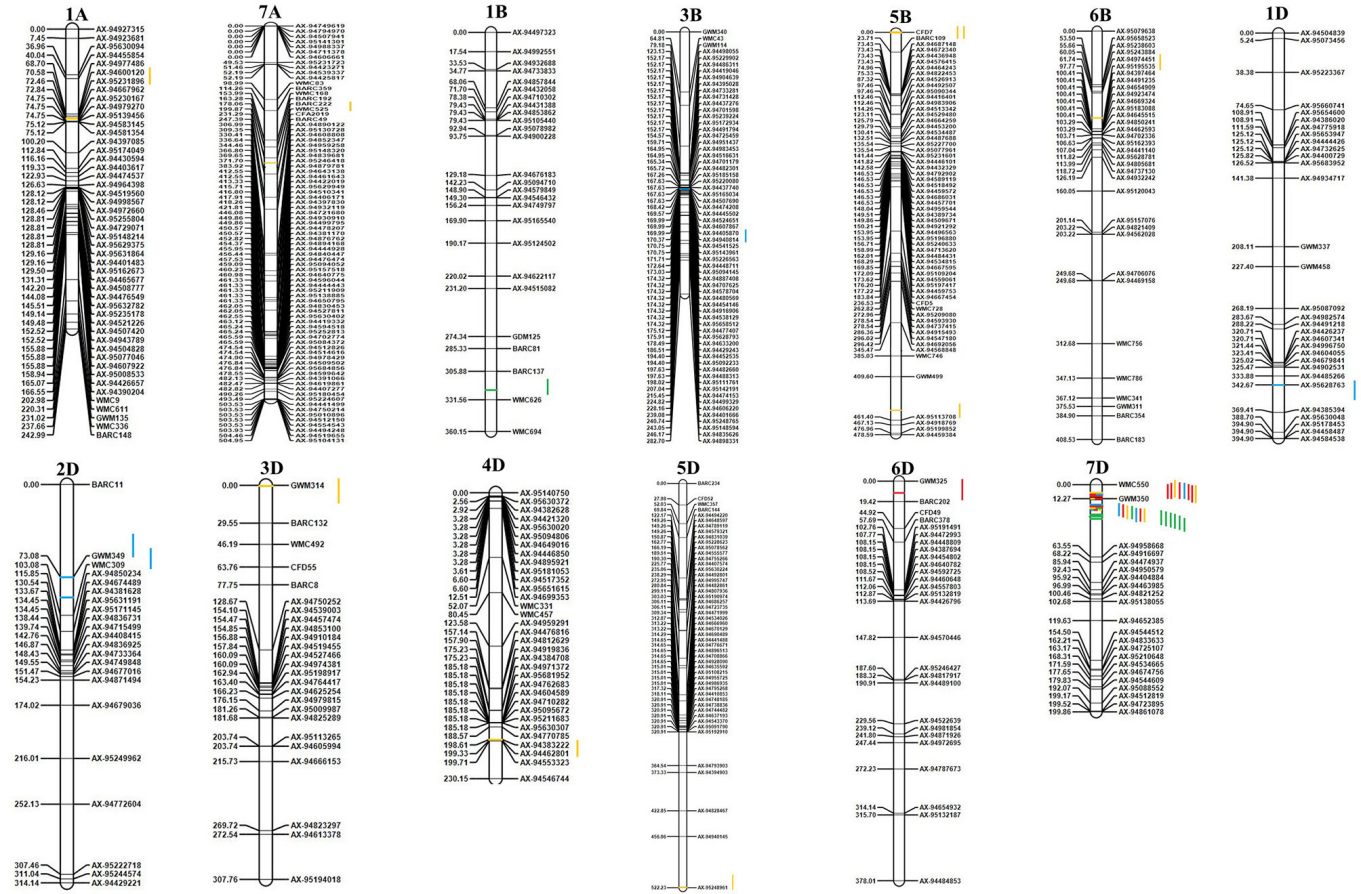
Genetic linkage map and QTL positions identified in A, B, and D genomes of RILs derived from WH 542 × synthetic derivative cross. Red color indicates QTLs for GFeC; Blue color indicates QTLs for GZnC; Yellow color indicates QTLs for grain GPC; Green color indicates QTLs for TKW.

The mapped QTL across the locations and years are presented in Table 4, and the linkage map with the identified QTL position is depicted in Figure 3. A total of 21 QTL were identified in 13 chromosomes representing all three genomes of wheat. Two, five, and 14 QTL were mapped on the A, B, and D genomes, respectively. Chromosome 7D represented the maximum number of seven QTL. A total of 21 QTL were mapped between 16 flanked regions (Table 4); the maximum number of four QTL was identified between flanking markers *Xgwm350–AX-94958668*, followed by three QTL between *Xwmc550–Xgwm350* in the7D chromosome. Trait-wise highest QTL were identified for GPC (10 QTL), followed by GZnC (six QTL), GFeC (three QTL), and TKW (two QTL). QTL for GFeC were mapped in chromosomes 6D and 7D; for GZnC in chromosomes 3B, 1D, 2D, and 7D; for GPC in chromosomes 1A,7A, 5B, 6B, 3D, 4D, 5D, and 7D and for TKW in chromosomes 1B and 7D.

**Table 4:** QTL identified for grain iron, zinc, protein, and thousand kernel weight in RIL population grown across three locations for 2 years.

**Trait**	**QTL name**	**Environment**	**Position**	**Flanking markers**	**LOD**	**PVE (%)**	**Add**	**Confidence interval**
GFeC	*QGFe.iari-7D.1*	ICAR-IARI_Y1	11	*Xwmc550–Xgwm350*	14.07	32.10	−1.22	7.5–18.5
		ICAR-IARI_Y2	12		17.92	42.13	−1.68	9.5–12.5
		GBPUAT_Y2	12		6.26	16.21	−1.28	6.5–24.5
	*QGFe.iari-7D.2*	GBPUAT_Y1	13	*Xgwm350–AX-94958668*	5.90	15.42	−0.55	7.5–27.5
		PusaBihar_Y1	21		6.73	16.00	−0.92	12.5–30.5
		PusaBihar_Y2	19		7.76	20.19	−1.16	8.5–30.5
		Pooled mean	13		18.48	37.44	−1.05	10.5–19.5
	*QGFe.iari-6D*	Pooled mean	11	*Xgwm32–Xbarc202*	2.57	5.61	−0.41	0–25.5
GZnC	*QGZn.iari-7D.2*	GBPUAT_Y1	21	*Xgwm350–AX-94958668*	3.58	13.07	−1.03	4.5–41.5
		Pooled mean	18		4.39	10.65	−0.80	6.5–35.5
	*QGZn.iari-2D.2*	Pooled mean	73	*Xbarc11–Xgwm349*	3.26	5.05	−0.56	52.5–87.5
	*QGZn.iari-2D.1*	ICAR-IARI_Y1	89	*Xgwm349– Xwmc309*	2.98	8.11	−1.23	73.5–104.5
	*QGZn.iari-3B*	ICAR-IARI_Y2	170	*AX-94405870– AX-94940814*	2.67	5.01	0.95	169.5–170.5
	*QGZn.iari-7D.1*	ICAR-IARI_Y2	11	*Xwmc550– Xgwm350*	2.97	6.05	−1.04	2.5–30.5
	*QGZn.iari-1D*	PusaBihar_Y1	343	*AX-95628763– AX-94385394*	2.55	5.28	−1.25	338.5–354.5
GPC	*QGPC.iari-5B.1*	ICAR-IARI_Y1	0	*Xcfd7– Xbarc109*	5.72	11.39	−0.35	0–7.5
		Pooled mean	0		2.60	4.76	−0.19	0–11.5
	*QGPC.iari-7D.2*	GBPUAT_Y2	13	*Xgwm350– AX-94958668*	2.57	4.67	−0.24	4.5–34.5
		PusaBihar_Y2	20		4.05	9.57	−0.57	9.5–31.5
		Pooled mean	21		3.38	12.91	−0.31	7.5–35.5
	*QGPC.iari-7A*	ICAR-IARI_Y2	185	*Xbarc222–Xwmc525*	3.37	10.53	−0.31	178.5–193.5
	*QGPC.iari-1A*	GBPUAT_Y1	72	*AX-94600120–AX-95231896*	2.87	4.73	−0.25	70.5–72.5
	*QGPC.iari-4D*	GBPUAT_Y1	199	*AX-94383222–AX-94462801*	4.09	6.71	0.31	194.5–199.5
	*QGPC.iari-5D*	GBPUAT_Y1	522	*AX-94940145–AX-95248961*	4.79	8.06	−0.33	508.5–522
	*QGPC.iari-5B.2*	GBPUAT_Y2	450	*Xgwm499–AX-95113708*	2.52	9.19	−0.33	430.5–465.5
	*QGPC.iari-6B*	PusaBihar_Y1	87	*AX-94974451–AX-95195535*	2.69	9.05	0.32	69.5–100.5
	*QGPC.iari-3D*	PusaBihar_Y1	0	*Xgwm314–Xbarc132*	3.24	6.33	0.26	0–8.5
	*QGPC.iari-7D.1*	PusaBihar_Y1	10	*Xwmc550–Xgwm350*	2.62	6.15	−0.26	2.5–31.5
TKW	*QTkw.iari-7D*	ICAR-IARI_Y1	30	*Xgwm350–AX-94958668*	6.70	21.05	−2.18	20.5–39.5
		ICAR-IARI_Y2	28		10.78	26.53	−3.75	19.5–37.5
		GBPUAT_Y1	20		5.73	11.02	−1.32	12.5–32.5
		GBPUAT_Y2	27		6.22	21.19	−2.61	10.5–41.5
		PusaBihar_Y1	27		7.01	23.12	−1.93	13.5–37.5
		Pooled mean	26		11.85	27.30	−2.17	18.5–35.5
		PusaBihar_Y2	27		8.84	21.89	−3.27	13.5–36.5
	*QTkw.iari-1B*	PusaBihar_Y2	323	*Xbarc137–Xwmc626*	2.68	4.22	1.45	311.5–336.5

*GFeC, grain iron concentration; GZnC, grain zinc concentration; GPC, grain protein content; TKW, thousand kernel weight; Y1: 2012–13; Y2: 2013–14. Positive value indicates that the allele was inherited from WH542, and negative value indicates that the allele was inherited from synthetic derivative*.

### Quantitative Trait Loci for Micronutrients

Three QTL governing GFeC were identified and are presented in Table 4. QTL governing GFeC explained 16–42.13% of the phenotypic variance. *QGFe.iari-7D.2*, flanked between *Xgwm350–AX-94958668*, was mapped in three environments (GBPUAT_Y1, PusaBihar_Y1, and PusaBihar_Y2) as well as in pooled mean, and contributed 20.19% to the phenotypic variance, followed by *QGFe.iari-7D.1* in three environments (ICAR-IARI_Y1, ICAR-IARI_Y2, and GBPUAT_Y2) and flanked between *Xwmc550–Xgwm350. QGFe.iari-7D.1* explained 42.13% of the phenotypic variance. Another QTL, *QGFe.iari-6D*, flanked between *Xgwm325–Xbarc202*, was mapped for pooled mean although it explained only 5.61% of the phenotypic variance. A total of six QTL were identified for GZnC and are presented in Table 4. QTL governing GZnC explained 5.01–13.07% of phenotypic variance. *QGZn.iari-7D.2*, flanked between *Xgwm350–AX-94958668*, was identified in GBPUAT_Y1 along with pooled mean and explained 13.07% of the phenotypic variance. *QGZn.iari-3B*, flanked between *AX-94405870–AX-94940814*, was identified at ICAR-IARI_Y2 and explained 5.01% of the phenotypic variance. *QGZn.iari-1D*, flanked between *AX-95628763–AX-94385394*, was mapped at Pusa Bihar_Y1 and explained 5.28% of the phenotypic variance. *QGZn.iari-2D.1* was identified in ICAR-IARI_Y1 with an explained phenotypic variance of 8.11% and flanked between *Xgwm349–Xwmc309*. *QGZn.iari-2D.2*, flanked between *Xbarc11–Xgwm349*, was mapped for pooled mean and explained only 5.05% of the phenotypic variance. *QGZn.iari-7D.1*, flanked between *Xwmc550–Xgwm350*, was mapped in ICAR-IARI_Y2 with 6.05% of the phenotypic variance.

### Quantitative Trait Loci for Grain Protein Content and Thousand Kernel Weight

Ten QTL governing GPC were identified and are presented in Table 4. QTL governing GPC explained 4.67% (*QGPC.iari-7D.2* at GBPUAT_Y2) to 11.39% (*QGPC.iari-5B.1* at ICAR-IARI_Y1). *QGPC.iari-7D.2*, flanked between *Xgwm350–AX-94958668*, was mapped in two environments (GBPUAT_Y2, Pusa Bihar_Y2) as well as pooled mean with 9.57% of the phenotypic variance, followed by *QGPC.iari-5B.1* at ICAR-IARI_Y1 and pooled mean with 11.39% of the phenotypic variance and flanked between *Xcfd7–Xbarc109*. The remaining eight QTL, i.e., *QGPC.iari-1A, QGPC.iari-7A, QGPC.iari-5B.2, QGPC.iari-6B, QGPC.iari-3D, QGPC.iari-4D, QGPC.iari-5D*, and *QGPC.iari-7D.1* were mapped in one environment each with an explained phenotypic variance of 4.73, 10.53, 9.19, 9.05, 6.33, 6.71, 8.06, and 6.15%, respectively. Two QTL governing the expression of TKW were identified in chromosomes 1B and 7D (Table 4). *QTkw.iari-7D* was identified at all the six tested environments and pooled means. It was flanked between *Xgwm350–AX-94958668* and explained 26.53% of the phenotypic variance. Another QTL, *QTkw.iari-1B*, was mapped at Pusa Bihar_Y2 and flanked between *Xbarc137–Xwmc626*. This QTL explained 4.22% of the phenotypic variance.

### Quantitative Trait Locus Additive Effects

The additive effects of the stable QTL were investigated for GFeC, GZnC, and GPC (Table 5). For the estimation of additive effects, we used all the novel and stable QTL identified in this study along with a stable genomic region identified in chromosome 2A in the previous study. For GFeC, *QGFe.iari-7D.2* had the largest effect individually, and there is no significant increase by combining the additional QTL and this QTL was identified in 31RILs. For GZnC, the two QTL combinations, *viz., QGZn.iari-2D.1* and *QGZn.iari-7D.1*, showed the highest average GZnC across the environments, and this combination was identified in eight RILs. For GPC, the four QTL combinations viz., *QGPC.iari-2A, QGPC.iari-5B, QGPC.iari-7D.1*, and *QGPC.iari-7D.2* showed the highest average GPC across the environments, and this combination was identified in three RILs.

**Table 5:** RILs with best combination of QTL for biofortification traits in wheat.

**QTL**	**Markers**	**Marker type**	**No. of RILs**	**1**	**2**	**3**	**4**	**5**	**6**	**Mean**
**Grain iron concentration**										
2A	*Xgwm249* + *Xgwm359*	B+B	45	42.1	41.1	36.7	38.6	35.3	35.7	38.3
7D.1	*Xwmc550* + *Xgwm350*	B+B	41	42.8	41.9	37.3	38.6	35.7	36.2	38.8
7D.2^*^	*Xgwm350* + *AX-94958668*	B+A	31	42.6	41.5	37.6	40.3	35.7	35.8	38.9
2A+7D.1	*Xgwm249 + Xgwm359* + *Xwmc550 + Xgwm350*	B+B+B+B	25	42.7	41.9	37.2	39.1	35.6	36.6	38.9
**Grain zinc concentration**										
2A	*Xgwm249* + *Xgwm359*	B+B	45	43	41.4	30.1	30	44.9	43.5	38.8
2D.1	*Xgwm349* + *Xwmc309*	A+A	20	43.4	40.8	29.9	30.4	45.1	42.9	38.8
7D.1	*Xwmc550* + *Xgwm350*	B+B	41	43.4	42.1	30.5	30.5	44.5	43.3	39.1
7D.2	*Xgwm350* + *AX-94958668*	B+A	31	43	42.4	30.8	30.8	43.4	43.1	38.9
2D.1+7D.1^*^	*Xgwm349* + *Xwmc309* + *Xwmc550* + *Xgwm350*	A+A+B+B	8	44.2	43.5	31.9	31.9	45.3	43.7	40.1
**Grain protein content**										
2A	*Xgwm249* + *Xgwm359*	B+B	45	16.4	14.6	14.4	13	16.7	16.4	15.3
5B.1	*Xcfd7* + *Xbarc109*	B+A	22	16.6	14.4	14.1	13	16.9	16.5	15.3
7D.1	*Xwmc550* + *Xgwm350*	B+B	41	16.6	14.8	14.7	13.3	16.9	16.5	15.5
7D.2	*Xgwm350* + *AX-94958668*	B+A	31	16.5	14.7	14.5	12.9	16.9	16.2	15.3
5B.1+7D.2	*Xcfd7* + *Xbarc109* + *Xgwm350* + *AX-94958668*	B+A+B+A	4	17.4	15.5	14.3	14.1	17.8	17.1	16.0
2A+5B.1+7D.1+7D.2^*^	*Xgwm249* + *Xgwm359* + *Xcfd7* + *Xbarc109* + *Xwmc550* + *Xgwm350* + *Xgwm350* + *AX-94958668*	B+B+B+A+B+B+B+A	3	17.8	15.8	14.6	14.3	18.2	17.2	16.3

* *Best combination of QTL, A—Parent 1 type, B—Parent 2 type*.

### *In silico* Analysis

The *in silico* analysis identified many important candidate genes underlying 10 QTL, with the highest PVE and pleiotropic for GFeC, GZnC, GPC, and TKW (Table 6). A pleotropic genomic region on chromosome 2A in our previous study (55) was also considered. Most significantly, QTL *QGZn.iari-2A, QGFe.iari-2A, QGpc.iari-2A, QGFe.iari-7D.2, QGZn.iari-7D.2, QGGpc.iari-7D.2, QTkw.iari-7D, QGFe.iari-7D.1, QGZn.iari-7D.1*, and *QGpc.iari-7D.1* were located in regions where genes coding for various transcription factors (TraesCS2A02G063800, TraesCS7D02G521500), transporters (TraesCS7D02G338100, TraesCS7D02G521400, and TraesCS7D02G338100), and signaling and catalytic molecules were present (TraesCS2A02G192400, TraesCS2A02G063900, TraesCS7D02G521700, TraesCS7D02G338400, and TraesCS7D02G521200) (Table 6).

**Table 6:** Putative candidate genes for grain iron (GFeC), zinc (GZnC), protein (GPC), and thousand kernel weight in the RIL population.

**QTL**	**Chr**.	**TraesID**	**Putative candidate genes (overlapping/nearby)**	**Molecular function**
*QGZn.iari-2A*	2A	TraesCS2A02G192500	Alpha/beta hydrolase fold	–
*QGFe.iari-2A*		TraesCS2A02G192400	GIY-YIG endonuclease	DNA binding
*QGpc.iari-2A*		TraesCS2A02G063800	Homeobox-like domain superfamily/SANT/Myb domain	DNA binding
		TraesCS2A02G063900	Protein kinase-like domain superfamily	Protein kinase activity, ATP binding
*QGFe.iari-7D.2*	7D	TraesCS7D02G337800	Reticulon-like protein	–
*QGZn.iari-7D.2*		TraesCS7D02G337900	Ribulose-phosphate binding barrel, N-(5′ phosphoribosyl) anthranilate isomerase (PRAI)	Catalytic activity, phosphoribosylanthranilate isomerase activity
*QGPC.iari-7D.2*		TraesCS7D02G338100	Aluminum-activated malate transporter	Malate transport
*QTkw.iari-7D*		TraesCS7D02G521800	WD40/YVTN repeat-like-containing domain superfamily, U3 small nucleolar RNA-associated protein	Protein binding
		TraesCS7D02G521700	RNA-binding S4 domain superfamily, Pseudouridine synthase, catalytic domain superfamily	RNA binding, pseudouridine synthase activity
		TraesCS7D02G521500	Zinc finger, MYND-type	–
		TraesCS7D02G521400	SWEET sugar transporter	Carbohydrate transport
		TraesCS7D02G521200	Serine/threonine protein kinase domain containing protein	–
*QGFe.iari-7D.1*	7D	TraesCS7D02G337800	Reticulon-like protein	–
*QGZn.iari-7D.1*		TraesCS7D02G337900	Ribulose-phosphate binding barrel, N-(5′ phosphoribosyl) anthranilate isomerase (PRAI)	Catalytic activity, phosphoribosylanthranilate isomerase activity
*QGPC.iari-7D.1*		TraesCS7D02G338100	Aluminum-activated malate transporter	Malate transport
		TraesCS7D02G338200	GAT domain superfamily, ENTH/VHS	Intracellular protein transport
		TraesCS7D02G338300	Domain unknown function DUF295	–
		TraesCS7D02G338400	Peptidase C78, ubiquitin fold modifier-specific peptidase 1/2	–

## Discussion

Genetic biofortification is the most cost-effective and sustainable strategy to control malnutrition. Understanding the genetic basis of complex traits like micronutrients, protein, and thousand kernel weight by QTL mapping will help in devising appropriate breeding strategies through MAS. The expression of all the studied traits in this study is greatly affected by the environment and GEI. Similar results of greater magnitude of the environment and GEI have been reported in previous studies for PC and TKW (18–20) and also for iron and zinc (13, 19, 59). Among the studied traits, GZnC was the most variable, whereas, TKW was the most stable. The lowest and highest pooled heritability was observed for GZnC and TKW, respectively, and a reverse trend was observed for CV (GZnC: 6.87; TKW: 4.57). Although both location and year effects were visible for all the traits, the magnitude of the location effect was found to be more pronounced than the year effect (Figure 1). The positive and significant associations among GFeC, GZnC, GPC, and TKW found in this study have also been reported in earlier studies (27, 29). In most of the earlier studies, the associations between GPC and TKW were negative. In this study, the associations between GPC and TKW were significantly positive in four out of six studied environments and non-significant negative in the Pusa Bihar_Y1 environment (r_g_ = −0.03), and non-significant positive in the Pusa Bihar_Y2 environment (r_g_ = 0.1). Similar results of both positive and negative associations between GPC and TKW have also been reported in some earlier studies (19, 27, 45, 60). The lowest and highest pooled heritability of GZnC and TKW, respectively, is also congruent with earlier studies (28, 44).

The linkage map was constructed with 836 high-quality informative markers (736 SNPs + 100 SSRs) and utilized for the QTL analysis. In the previous study conducted on the same population, the SSR-based genetic map had a very low frequency of markers in the D genome (55). As a result, none of the QTL is localized in the D genome. The addition of SNPs improved D genome marker density and distribution, particularly in the 7D chromosome. Enrichment of genetic linkage map with SNPs greatly helped in the mining of novel genomic regions in the D genome. As a result, a maximum number of novel QTL were also identified in the D genome (14 QTL).

The D genome generally shows a low level of polymorphism in naturally occurring hexaploid bread wheat due to its well-known evolutionary history and low recombination during its post-evolution era (61, 62). For this reason, synthetic hexaploid wheats (SHWs) were created by crossing tetraploid durum wheats with multiple accessions of *Ae. tauschii* (the D genome donor), which increased the diversity of the D genome (63–65). Studies have shown that the D genome diversity of SHW is considerably greater than that of bread wheat (66, 67). In this study, since a synthetic parent was involved in the cross, D genome polymorphism improved significantly and 25.1% (210) of the markers mapped on the D genome (Table 3). A similar trend in marker distribution has been observed in earlier studies involving SHWs as one of the parents in creating mapping populations (30).

A total of 21 QTL were identified in 13 chromosomes representing all three genomes of wheat. Intriguingly, the alleles at most of the QTL responsible for increased GFeC, GZnC, GPC, and TKW were inherited from the synthetic derivative parent. Three QTL (*QGFe.iari-6D, QGFe.iari-7D.1*, and *QGFe.iari-7D.2*) governing GFeC were identified in chromosomes 6D and 7D. Also, in the earlier study, grain iron QTL have been identified in chromosome 7D (34) with different marker intervals, whereas QTL (*QGFe.iari-6D*) mapped on 6D in this study is novel and not reported by earlier studies. For grain zinc, a total of six QTL (*QGZn.iari-2D.1, QGZn.iari-3B, QGZn.iari-7D.1, QGZn.iari-7D.2, QGZn.iari-1D*, and *QGZn.iari-2D.2*) were identified. The localization of QTL for GZnC reported in earlier studies on 3B (30, 39), 1D (39), 2D (39), and 7D (30) in different mapping populations corroborate the involvement of these chromosomes.

For GPC, 10 QTL were identified and designated as *QGPC.iari-5B.1, QGPC.iari-7A, QGPC.iari-1A, QGPC.iari-4D, QGPC.iari-5D, QGPC.iari-5B.2, QGPC.iari-7D.1, QGPC.iari-6B, QGPC.iari-3D*, and *QGPC.iari-7D.2*. The association of genomic regions for GPC in chromosomes 1A (42, 68), 5B (36, 47), 6B (36, 42), 3D (45), and 5D (45, 47) was also reported in previous studies. Additionally, five novel QTL were identified in 7A (*QGPC.iari-7A*), 4D (*QGPC.iari-4D*), 5D (*QGPC.iari-5D*), and 7D (*QGPC.iari-7D.1* and *QGPC.iari-7D.2*), which were missing in the earlier studies. Interestingly, one novel QTL (*QGPC.iari-7D.2*) was also found to be stable. There were two QTL (*QTkw.iari-1B* and *QTkw.iari-7D*) identified in chromosomes 1B and 7D governing TKW. The genomic regions associated with TKW in these two chromosomes have also been reported in the previous studies (40, 42).

In the earlier study, a total of 16 QTL were identified including four QTL for GFeC, five QTL for GZnC, two QTL for GPC, and five QTL for TKW. The QTL together explained 20, 32, 24.1, and 32.3% of the phenotypic variance, respectively, for GFeC, GZnC, GPC, and TKW. In contrast to the earlier study, where the D genome was completely missing, this study identified the majority of QTL from the D genome. This is due to fairly good marker coverage in all the three genomes in this study, unlike the earlier study, wherein, marker coverage in the D genome was very sparse. The total phenotypic variance explained for all the QTL for any given trait except TKW was higher in this study compared with the earlier studies. Similarly, for two traits, i.e., GFeC and TKW, the highest explained phenotypic variance for an individual QTL was higher compared with the earlier identified QTL. The highest explained phenotypic variance for an individual QTL was 42.13% for GFeC and 26.53% for TKW compared with the earlier identified QTL with 6.8 and 10.4%, respectively.

The environment and GEI play a key role in the expression of quantitative traits. Identification of stable genotypes with the high buffering ability and of QTL is of paramount importance to use in breeding programs. Genetic dissection of complex traits by the identification of stable QTL will complement varietal development by molecular breeding approaches. In this study, four stable QTL (*QTkw.iari-7D, QGFe.iari-7D.2, QGFe.iari-7D.1*, and *QGPC.iari-7D.2*) were identified in two or more environments. *QTkw.iari-7D* was identified in all the six tested environments and pooled mean, followed by *QGFe.iari-7D.2* and *QGFe.iari-7D.1*, which were identified in three environments along with pooled mean. Stable QTL identified in more than two environments were also reported for GPC and TKW (42, 45), GPC (39, 47, 68), GFeC and GZnC (30), and GFeC (33, 37).

Identification of the best combination of QTL effects by estimation of additive effects of the stable QTL will provide an opportunity to utilize RILs with the best combination as donors. The best combination of QTL for all the three biofortification traits in RILs was identified. There is no additional advantage of additive QTL over the individual QTL effects in the expression of GFeC. However, the QTL combination of the two QTL combinations, *viz., QGZn.iari-2D.1* and *QGZn.iari-7D.1*, showed the highest average GZnC across the environments, and this combination was identified in eight RILs. For GPC, the four QTL combinations, viz., *QGPC.iari-2A, QGPC.iari-5B.1, QGPC.iari-7D.1*, and *QGPC.iari-7D.2* showed the highest average GPC across the environments, and this combination was identified in three RILs. Although numerically additive QTL effects for GZnC and GPC are higher than the individual QTL effect, statistically they are at par.

Genomic regions harboring co-located QTL for two or more traits were also identified. This information is helpful in the simultaneous improvement of multiple traits without many additional interventions. Two common genomic regions associated with different co-localized QTL governing two or more traits were identified in chromosome 7D where the genomic region flanked between *Xgwm350–AX-94958668* was associated with the maximum number of four co-localized QTL (*QGFe.iari-7D.2, QGZn.iari-7D.2, QGPC.iari-7D.2*, and *QTkw.iari-7D*). Another region flanked between *Xwmc550–Xgwm350* was also associated with three co-localized QTL (*QGFe.iari-7D.1, QGZn.iari-7D.1*, and *QGPC.iari-7D.1*). Some of the other studies (13, 15, 16, 37–40) have also identified such pleiotropic region(s) associated with two or more traits, namely, GFeC, GZnC, GPC, and TKW. High positive correlations observed in this study also strongly support the co-localization of genomic regions governing GFeC, GZnC, GPC, and TKW. Only few studies have reported the association of TKW in the same region as GPC or even with GZnC and GFeC (42, 44, 45, 68). All these studies reported a positive correlation for GPC and TKW. Considering the positive correlations obtained between TKW and GPC in all the environments, except Pusa Bihar_Y1, in this study, it was not surprising to find such pleiotropic QTL (in 2A and 7D chromosomes). The co-location of GFeC, GZnC, and GPC is well documented. For example, the *Gpc-B1* locus derived from *T dicoccoides* is effective in improving GFeC, GZnC, and GPC by 18, 12, and 38%, respectively (15, 16).

The *in silico* BLAST search identified various potential candidate genes underlying QTL with high PV or pleiotropic QTL for GZnC, GFeC, GPC, and TKW (Table 6). Various QTL identified in chromosomes 2A and 7D were located in regions where gene coding for transcription factors, transporters, and kinase-like superfamilies was present. For example, the SANT domain (coded by *TraesCS2A02G063800*) is generally found in combination with domains of Zn finger type transcription factors, such as the C2H2-type and GATA-type transcription factors, the role of which has been suggested to be in Zn uptake and homeostasis in plants (69, 70). Members of serine-threonine/protein kinase-like superfamilies are known to catalyze phosphorylation processes, thus controlling growth and development, and some are known to activate Zn channels and transporters (71). The well-characterized serine/threonine-protein kinase encoding gene in maize (*KNR6*; kernel number per row: six) has been shown to determine the kernel number and ear length in maize (72). Since both maize and wheat are members of the *Poaceae*/*Gramineae* family, it would be interesting to further investigate the functional role of the serine/threonine-protein kinase genes identified here (coded by *TraesCS7D02G521200* and *TraesCS2A02G063900*).

In the past decade, the role of various transporters has been shown in regulating mineral homeostasis in plants. These transporters play critical roles in the transport of small peptides, secondary amino acids, glutathione conjugates, and mineral uptake. Many of these transporters have proven to be involved in long-distance iron transport or signaling in Arabidopsis (73). In this regard, an important role of the aluminum-activated malate transporter (ALM1), in combination with a Zn finger-type transcription factor (STOP1), has been shown in regulating iron homeostasis in Arabidopsis (74). In both the *stop1* and *almt1* mutants, the accumulation of Fe in the root apex was found to be greatly reduced (75).

## Conclusion

We earlier reported QTL for different biofortification traits, *viz*., grain zinc, iron, protein, and thousand kernel weight utilizing an SSR-based genetic map of 286 RIL population developed between a cultivated bread wheat variety and a synthetic derivative. In this study, we added 736 informative SNPs and analyzed a smaller subset of the same population for these traits. New QTL were identified in this study, and many of these were found located in the D genome. The co-localization of QTL for different traits was also observed. Chromosome 7D, in particular, harbored seven and three co-localized QTL at different positions. This indicates that at least some common pathways may be involved in the uptake or accumulation of the micronutrients. Several consistent QTL over two or more environments for different traits are identified in this study as well. Best QTL combinations in RILs have been identified through additive effects, and these combinations would be potential donors to be utilized in future breeding programs. Furthermore, the identification of pleiotropic regions for GZnC, GFeC, GPC, and TKW suggests the possibilities for genetic improvement of GZnC and GFeC without compromising grain yield and GPC. Further fine mapping to identify linked or functional markers is envisaged.

## Data Availability Statement

The datasets presented in this study can be found in online repositories. The names of the repository/repositories and accession number(s) can be found in the article/Supplementary Materials.

## Author Contributions

AS conceptualized the investigation. Field experimentation at Delhi location was conducted by AS, AA, GS, and SaS at Bihar location by IS and RS, at Pantnagar by JJ and GK. Genotyping was done by SuS and GK. Statistical analysis including QTL mapping was done by NDR and DS. Original draft was prepared by GK, AS, and NDR. Review and editing was done by AS, DS, and GK. All authors contributed to the article and approved the submitted version.

## Conflict of Interest

The authors declare that the research was conducted in the absence of any commercial or financial relationships that could be construed as a potential conflict of interest.
